# Strategies for HIV-1 suppression through key genes and cell therapy

**DOI:** 10.3389/fmed.2023.1259995

**Published:** 2023-11-29

**Authors:** Alyona Sorokina, Elizaveta Anchakova, Erdem Dashinimaev

**Affiliations:** ^1^Center for Precision Genome Editing and Genetic Technologies for Biomedicine, Pirogov Russian National Research Medical University, Moscow, Russia; ^2^Moscow Institute of Physics and Technology (State University), Dolgoprudny, Russia

**Keywords:** HIV-1, AIDS, cART, CCR5, CRISPR/Cas9 findings number

## Abstract

Human immunodeficiency virus type 1 (HIV-1) remains a significant challenge for global public health as limited therapeutic options are available for HIV-infected individuals receiving combination antiretroviral therapy. Additionally, individuals with HIV-1/acquired immunodeficiency syndrome (AIDS) complications have a reduced life expectancy. In recent decades, gene and cell-based strategies have shown promise in achieving a functional cure for HIV-1 infection. The outcomes of therapies with patients in Berlin and London have led to moderate optimism for a highly effective HIV-1 treatment. This review categorizes current strategies for HIV-1 treatment into RNA- and antibody-based therapies, cell and genome editing approaches, and methods for eradicating latent reservoirs. These findings demonstrate how the use of various anti-HIV-1 agents enhances our understanding of HIV-1 infection and may provide important insights for potential HIV-1 treatment.

## 1 Introduction

Human immunodeficiency virus type 1 belongs to the family *Retroviridae* of the genus *Lentivirus*. Two main types of the human immunodeficiency virus (HIV) exist, with HIV-1 being the first one discovered and the most common type found globally, whereas HIV type 2 is primarily found in West Africa (1). The viral genome (approximately 9.8 kb) consists of nine open reading frames that encode 15 proteins and govern all processes involved in the virus life cycle, including receptor binding, membrane fusion, reverse transcription, integration, protease processing, and virus assembly (2). The HIV-1 RNA genome contains structural genes (*pol*, *gag*, *env*), regulatory genes (*tat*, *rev*, *vpr*, *nef*), and accessory genes (*vpu*, *vif*) (3, 4).

Scientific advancements suggest that the threat of an HIV pandemic to public health may be eliminated by 2030, but currently, HIV-1 remains one of the most significant problems for global public health (5). HIV-1 causes acquired immunodeficiency syndrome (AIDS), and HIV-infected patients often suffer from opportunistic infections, cardiovascular and neurological diseases, as well as AIDS-related illnesses such as Hodgkin’s disease, non-Hodgkin’s lymphoma, lymphocytic leukemia, and other malignant and non-malignant complications that shorten the lifespan (4, 6, 7).

Approximately 79.3 million people have been infected with HIV-1 since the beginning of the pandemic, and approximately 50% of them have died from AIDS, according to statistics from the World Health Organization. In 2020, an estimated 1.5 million people were newly infected, and around 690,000 people died from illnesses related to AIDS, according to the Joint United Nations Programme on HIV/AIDS (UNAIDS) (8).

Existing methods for treating HIV-1 can be divided into two types: sterilizing and functional. The sterilizing method involves the complete elimination of replicative HIV-1 proviruses from the human body. The functional method, on the contrary, aims to control the replication of HIV-1 in the long term and maintain a normal level of CD4 + T cells, despite the presence of hidden HIV-1 reservoirs (hidden integrated provirus) and the absence of antiretroviral therapy (9–11).

Ideally, preventive HIV vaccines should prevent HIV infection by ensuring sterilizing immunity via stimulation of high titers of broadly neutralizing antibodies (12). Although such sterilizing agent is still far from available, several efforts have been made toward achieving functional cure of HIV-1 infection.

Currently, the standard treatment for HIV infection, known as combination antiretroviral therapy (cART), involves regular administration of a combination of antiretroviral drugs that block various stages of the virus replication cycle (13). Standard cART can greatly reduce the amount of virus in the blood of HIV-infected patients (viremia) to levels that cannot be detected and turn a fatal infection into a chronic disease that can be medically controlled (4, 14).

Despite the remarkable efficacy of cART, there are still several issues: (a) cART is unable to eradicate the hidden reservoir; (b) patients rely on daily and strict adherence to the treatment regimen; (c) potential side effects after negative drug interactions may occur; (d) cART can lead to drug resistance and limited therapeutic options in multi-class resistant HIV infection; (e) in some countries, this therapy is economically inefficient, and some individuals have limited access to antiretroviral drugs; and (f) there are limitations due to social issues, such as “social stigma.” Also, this therapy has side effects, such as chronic inflammation, CVD, frailty, etc. (15–17).

Therefore, in addition to cART, other promising approaches to achieving functional cure of HIV-1 have been studied in recent decades. Initially, gene therapy methods used RNA interference (RNAi) to suppress the expression of viral mRNA or host mRNA required for HIV-1 infection. This approach allowed for the development of sequence-specific agents to compensate for viral mutations, significantly expanding the number of therapeutic options beyond cART (18). With the passage of time, the technologies underlying the initial clinical trials of gene therapy for HIV-1 have been complemented by alternative gene therapy options such as the utilization of programmable nucleases. Clustered Regularly Interspaced Short Palindromic Repeat (CRISPR)/CRISPR-associated protein 9 (Cas9) systems, when compared to other programmable nucleases, provide enhanced efficacy and simplicity, and the capacity to execute multiplex genome engineering to target different stages of the virus’s life cycle (19, 20). The combination of CRISPR-Cas9 therapy and cell therapy based on CCR5-depleted hematopoietic stem cells (HSCs), in an attempt to recreate the CCR5Δ32 mutation, could be a valuable addition to the existing spectrum of cART (21).

**FIGURE 1:**
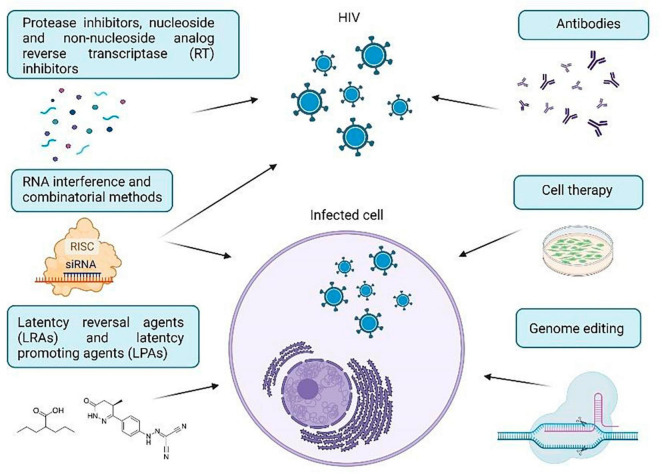
Current strategies for HIV-1 treatment.

The purpose of this review is to investigate certain important techniques for treating HIV-1 using approaches in cell and gene therapy. We will provide a brief overview of the molecular mechanisms utilized in therapeutic approaches to combat HIV-1, commonly used anti-HIV drugs, viral and cellular targets for HIV therapy, and the current challenges in the use and delivery of anti-HIV agents. HIV-1 treatment strategies have been categorized into RNA therapy, antibody-based therapy, cell therapy, genome editing strategies, and methods used to eradicate hidden reservoirs (Figure 1). Taken together, these findings demonstrate how the implementation of various agents advances our comprehension of HIV-1 infection and can offer significant insights for future HIV-1 treatments.

## 2 RNA therapeutic strategies

### 2.1 RNA interference

The canonical pathway of RNA interference is initiated by the recognition and cleavage of intracellular long double-stranded RNA (dsRNA) intermediates or endogenous microRNAs (miRNA) into small interfering RNAs (siRNAs) of 21-23 nucleotides by the endoribonuclease Dicer (22, 23). The Argonaute protein, specifically Ago2 in mammals, plays a crucial role in the RNA-induced silencing complex (RISC) as an important component. One of the siRNAs becomes loaded onto Ago2, and RISC then utilizes the guide strand of the loaded siRNA to identify and cleave the mRNA that is complementary to it in the middle of the siRNA:mRNA duplex. Meanwhile, the non-targeted (passenger) strand of the loaded siRNA is eliminated (24, 25). Formation of the miRNA:mRNA duplex leads to the degradation of the mRNA fragment by cellular exonucleases (26). Besides, the canonical RNAi pathway functions in the cytoplasm, RNAi can also cause suppression of gene-specific transcription in the nucleus (18).

Anti-HIV-1 RNAs can be separated into two main groups according to their molecular targets: one group uses antisense mechanisms to target viral RNA or host factors, while the other group consists of RNA decoys and aptamers targeting proteins and acting as steric or competitive inhibitors (27, 28). There are different strategies to combat HIV-1 RNA, which use various active agents. Some of these agents include antisense oligonucleotides, small nuclear ribonucleoproteins U1, ribozymes, endoribonucleases, RNA aptamers, small RNA duplexes such as microRNAs, siRNAs, substrate RNA Dicer, short hairpin RNAs, and Ago/shRNAs (29–31).

Synthetic mature siRNA or short shRNA can be used to create artificial dcrRNA, which can be transfected into the cell, or synthetic miRNA can be synthesized, which can be expressed intracellularly from a transgenic construct (32). It is important to note that there are no miRNAs that are currently known to have anti-HIV properties, although some research groups have explored potential molecular candidates for this purpose (33). The therapeutic strategy known as “block and lock” also involves a more in-depth discussion of long non-coding RNAs (lncRNAs).

The most frequently used engineered miRNAs consist of two 21-nucleotide RNA strands with a two-nucleotide overhang at the 3′ end of every single chain (27). Previous studies indicate that dsRNA designs that require Dicer cleavage are more efficient than conventional siRNAs that have been developed based on sequence (34, 35). For the effective functioning of siRNA RISC, it is necessary to choose the right guide chain, as well as to ensure high thermodynamic stability of the siRNA ends, optimal G/C content and the absence of immunostimulating sequences in order to lower the chance of side effects (occurrence) (36–38). short hairpin RNAs (shRNAs) are synthetic oligonucleotides that contain a siRNA sequence followed by a 9-nucleotide loop and a sequence complementary to the siRNA sequence (39, 40). After being exported from the nucleus to the cytoplasm by Exportin-5, shRNA triggers the mechanism of RNAi. Additionally, it has been observed that shRNAs are 10 times more active than siRNA (41). A database named HIVsirDB is available for free and can predict the effectiveness of miRNAs against HIV-1. It encompasses 26 different strains of HIV and 28 different types of cells (42). Hammerhead or hairpin ribozymes can be constructed with ease, and unlike RNAi molecules, they do not depend on the presence of cellular factors such as Exportin-5, Drosha, or Dicer for processing or cleaving complementary RNA target sequences. They are also similar to aptamers in this regard (43). MazF endoribonuclease, derived from Escherichia coli, cleaves single-stranded RNA at 5′-ACA positions, providing an attractive tool for targeting HIV-1 RNA (31).

Single-stranded aptamers inhibit the activity of HIV-1 by directly interacting with the key proteins required for the virus replication cycle. They are generated from random sequence RNA libraries by an iterative selection and amplification procedure known as SELEX (Systematic Evolution of Ligands by EXponential enrichment) (44). In addition, there are ASOs, which are also single-stranded synthetic nucleic acids that bind to mRNA through base pairing and either induce degradation of their targets through an RNase-dependent mechanism or they can interrupt mRNA splicing or translation through a mechanism of steric blocking (45).

U1 snRNP, an important element of the splicing mechanism, is considered as a possible agent for the cure of the HIV-1 gene due to its composition of seven main proteins (SNRPB, SNRPD1, SNRPD2, SNRPD3, SNRPE, SNRPF, and SNRPG), which are assembled into a heptamer ring to prevent polyadenylation (4).

#### 2.1.1 Cellular and viral targets

Potential targets for therapy include structural genes (*Pol*, *Gag*, *Env*), regulatory genes (Tat, Rev, Vpr, Nef), and accessory genes (Vpu, Vif). These sites, including non-translated long terminal repeats (LTRs), may be targeted at spliced and non-spliced transcripts (18). Most strategies rely on manipulating RNAi to target viral transcripts in the cytoplasm, but the HIV-1 promoter can also be targeted to suppress gene transcription, leading to epigenetic silencing of the integrated provirus (26, 46).

**FIGURE 2:**
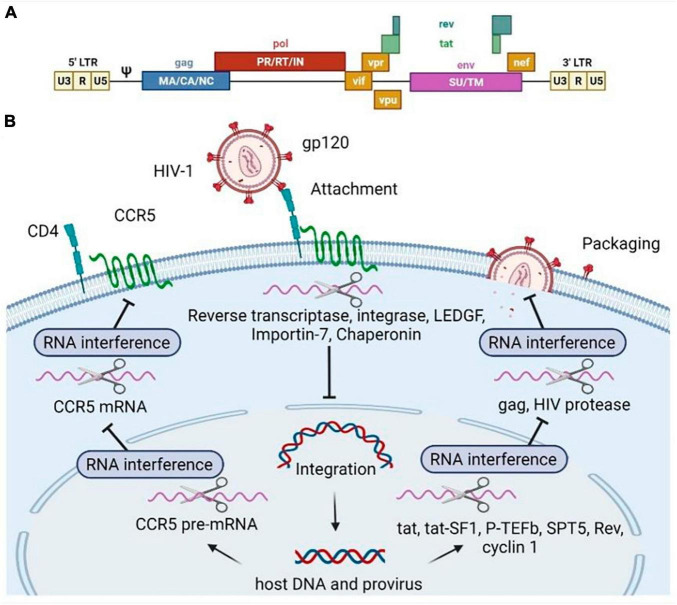
Human immunodeficiency virus type 1 (HIV-1) genome **(A)** and RNA-based inhibitors targeting various stages of the HIV-1 replicative cycle **(B)**. Scissors illustrate RNA molecules. The RNAs can specifically bind to the mRNA encoding the host (pTEFb, tat-SF1, SPT5, cyclin T1, CCR5, LEDGF, Importin-7, Chaperonin) factors or HIV genes and regulatory regions (gag, rev, pol, tat), then the target mRNA degradation is initiated. Pol, polymerase enzyme; Gag, group-specific antigens; Env, envelope surface glycoprotein gp160, precursor; Tat, *trans-*activator of transcription; Rev, regulator of expression of virion proteins; Vpr, viral protein R; Nef, negative regulatory factor; Vpu, viral protein U; Vif, viral infectivity factor; PR, pol includes protease; RT, reverse transcriptase; IN, integrase. Gag consists of MA, matrix; CA, capsid, NC, nucleocapsid; p6 domains (not shown). The Env spike protein has a surface part (SU) and transmembrane (TM) part. CCR5 is known as C-C chemokine motif receptor type 5.

Experiments have proved that siRNAs and shRNAs can be employed to target virtually all HIV-1 RNAs either upon viral uncoating or upon transcription from the proviral DNA (Figure 2B) (47, 48). The use of shRNAs or siRNAs that are complementary to the target gene or LTR can lead to significant reduction in the expression of viral proteins, thus ensuring protection of HIV-1 sensitive cells, including CD4 + T-cells, macrophages, monocytes and dendritic cells, through post-transcriptional gene silencing (49–52). For instance, inhibition of anti-HIV-1 miRNA transcription, constructed against *gag* and *env* in the viral genome (Figure 2A), has been detected in CD4-positive cells (3, 53). Moreover, hammerhead ribozymes targeting the HIV U5 and *pol* regions have been developed for the protection of Jurkat T-cell lines and PBMCs against both laboratory strains and clinical isolates of HIV (48). An RNA hammerhead ribozyme (Rz2) targeting overlapping vpr and tat ORFs of HIV-1 has also been developed and tested in macrophages, T-cell lines, and primary T-cells (54). The HIV-1 encapsidation signal (Ψ) is also an attractive target for gene therapy based on antisense RNA (55). Moreover, HIV genes encoding structural proteins (*Gag*, *Pol*, *Nef*, *Tat*) have often been considered as targets for RNAi-based gene therapy in clinical trials (26). Several early trials modified the viral regulatory proteins Rev and Tat to act as transdominant negative factors, in other words, these proteins blocked the export of viral RNA from the nucleus of infected cells (56). Zhu et al. conjugated an anti-CD4 aptamer with an siRNA molecule to target HIV-1 protease mRNA (Figure 2B) (57).

Targeting host cell cofactors required for HIV-1 infection is a promising strategy for RNAi therapy because these endogenous genes are less susceptible to mutational escape compared to viral genes, which are prone to error-prone reverse transcription (18). However, cellular targets must be carefully evaluated as these molecules affect both cell growth and function, and also because they may be significant for transmitting cell signals that are not obvious *in vitro* (26).

The binding of the viral protein gp120 to the cellular receptor CD4 and its co-receptors CCR5 and CXCR4 initiates membrane fusion and subsequent cell entry by HIV-1 (58). Therefore, the CD4 receptor, and the entry coreceptors CCR5 and CXCR4 have been considered attractive targets for shRNA therapy to prevent the initiation of HIV-1 infection and inhibit the fusion of the host cell membrane with HIV-1 (see Figure 2B) (28, 39). For example, one of the effective shRNAs targeting the R region of HIV-1 LTR was validated in a humanized mouse model in combination with shRNA targeting CCR5 mRNA (54). Previously, Eekels et al. used multiple shRNAs targeting 30 human genes involved in HIV-1 replication and identified TRBP, ALIX, and AGT6 as the most suitable genes for long-term inhibition of HIV-1 replication with minimal toxicity in shRNA-transduced T lymphocyte cells (59). Several human proteins (cyclin T1, SOCS1, and RNA helicase DDX3), including co-factors of viral integrase (LEDGF/p75, importin-7, and chaperonin), elongation factors (P-TEFb, Tat-SF1, and SPT5), were also mentioned as promising candidates for RNA-based therapy (Figure 2B) (18).

#### 2.1.2 Current status

Studies have shown that effective gene suppression is achieved only with a small number of RNA agents (13). RNAi may exhibit imperfection in RNA-RNA duplex formation, leading to off-target effects on unrelated mRNA (60). Additionally, if the passenger strand of the RNA molecule is loaded into RISC instead of the guide strand, it can cause RNAi side effects (37, 61).

Currently, the main problem is the delivery of RNA substances (as well as DNA plasmids, mini-circles and therapeutic transgenes) to infected cells or HIV-sensitive ones. Delivery systems play a fundamental role in facilitating the cellular uptake of RNA molecules and protecting them from nucleases degradation, thus minimizing the need for any chemical modifications that may alter the specificity and functionality of RNA (62, 63). Despite the successful application of anti-HIV-1 RNAi *in vitro*, current delivery methods have not been able to translate these achievements to *in vivo* conditions. Therefore, non-specific delivery methods or *ex vivo* methods such as electroporation, viral vectors, and nanoparticles are often used (18). Other methods of delivering target RNA include tissue-specific adeno-associated viruses (AAVs), nucleic acid aptamers, antibodies, and nanoparticles composed of cationic polymers (such as poly-L-lysine, polyethylenimine, polyamidoamine chitosan) and lipids. However, each of these approaches has its own challenges that require consideration in order to achieve optimal delivery solutions (18, 64).

### 2.2 RNA-vaccines

An mRNA vaccine is a synthetic vaccine that uses a DNA template to transcribe mRNA, which in turn triggers an immune response to the target pathogen. mRNA vaccines can be designed to express almost any antigen sequence, and the innate immune system’s ability to recognize viral RNA sequences enables the effective elicitation of an innate response along with the generation of cytokines and chemokines, that are crucial for a successful adaptive immune response (65, 66). It has also been shown that COVID-19 vaccines based on the mRNA platform have excellent immunogenicity and are able to stimulate B-cell and T-cell responses (67).

mRNA vaccines can be divided into several types, including self-amplifying mRNA vaccines (SAM), DC-mRNA vaccines, non-replicating mRNA vaccines, and mRNA vaccines against cancer. SAM vaccines create auxiliary equipment for the formation of double-stranded RNA and intermediates for replication and other products, and do not require mechanisms for replicating their RNA after introduction into cells (68, 69). They require a less dose to elicit a better immune response compared to non-amplifying RNA vaccines. SAM vaccines can be engineered by single-stranded positive-sense alphaviruses and delivered as virus replicon particles (VRP). mRNA is delivered through different strategies such as electroporation, cationic liposomes, or cationic Nano emulsion, and it carries the code for an RNA-dependent RNA polymerase together with the immunogen, which collectively contribute to a lasting immune response. The immune response can be measured by the number of Th1-type T cells generated after vaccination (70). All mRNA vaccines share common structural elements: cap, 5′UTR, 3′UTR, ORF, and poly(A) tail. They are generated through enzymatic transcription of a DNA template, and to create a vaccine, it is just required to change the sequence encoding the target antigen (71, 72).

#### 2.2.1 Delivery to cells

Delivery of mRNA vaccine into antigen-presenting cells (APCs) is a limiting factor for vaccine efficacy (71). mRNA vaccine against HIV, containing free mRNA, faces challenges in stability preservation and penetration into the cytoplasm of APCs during transportation. Various strategies have been employed to enhance the delivery of HIV mRNA vaccine into APCs (73). The method of vaccine administration influences the immune response and mRNA uptake. Utilizing different strategies, such as mRNA delivery into dendritic cells (DCs) or through nanoparticle carriers, can improve mRNA penetration into APC cytoplasm and induce a high level of immune response, holding promise for the development of effective HIV vaccines (74–76).

#### 2.2.2 Current status

Recent advancements in mRNA technology have led to increased utilization of vaccination experiments against HIV using improved delivery methods. However, clinical trials conducted on therapeutic vaccines against HIV-1 did not demonstrate significant clinical impact, despite achieving safety and efficacy in eliciting an immune response (77, 78).

### 2.3 CRISPR/Cas9

A variety of CRISPR-Cas systems have been identified, though only a select few have been leveraged as instruments for scientific research (20). Class 1 systems deploy a complex of multiple Cas proteins, while Class 2 systems utilize a single effector Cas protein (79, 80). The CRISPR-Cas9 system is most commonly utilized within human cells. The two-component RNA system, made up of CRISPR RNA (crRNA) and *trans-*activating crRNA (tracrRNA), has been streamlined into a single guide RNA (sgRNA) that aligns with a specific target sequence (81). For Cas9 to bind and cleave DNA, a protospacer adjacent motif (PAM) sequence is necessary; it lies downstream from the target sequence and results in either blunt or staggered double-strand breaks (DSBs) (20). Either homology-directed repair (HDR) or non-homologous end joining (NHEJ) can be employed for gene modification within mammalian cells (82).

**FIGURE 3:**
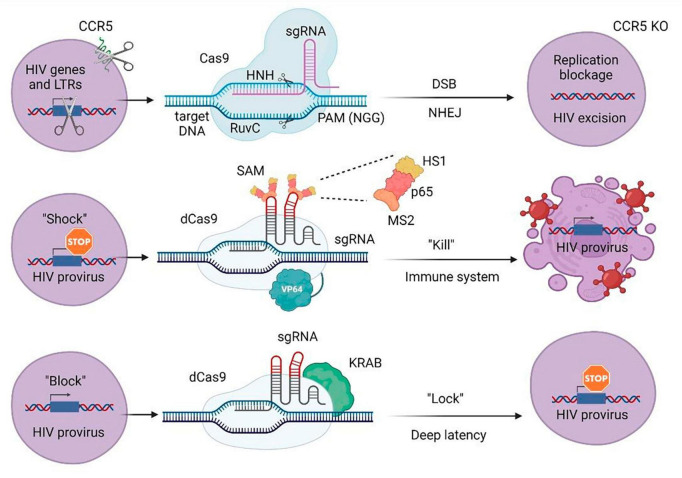
CRISPR/Cas approaches to attack HIV-1 or host-specific genes participating in the life cycle of the virus. LTRs, long terminal repeats; sgRNA, single guide RNA; PAM, protospacer adjacent motif; DSB, double stranded break; NHEJ, non-homologous end joining; KO, knockout; SAM, synergistic activation mediator; KRAB, Krüppel-associated box repressor; dCas9, nuclease-deactivated Cas9. NHN and RuvC are Cas9 catalytic domains that cut single-stranded DNA at the target site. MS2 protein via designed aptamers binds to the dCas9/VP64 fusion protein and involves additional activation domains (HS1 and p65).

A significant objective in combatting HIV-1 using the CRISPR/Cas9 strategy is to reduce or disable intact proviral sequences of HIV-1 (4). The fusion protein deficient in Cas9 (dCas9), in combination with sgRNAs targeting specific effector domains of DNA sequences, has been employed for gene activation or suppression of transcription (Figure 3) (83). Genome engineering supports the advancement of cell therapy, including a universal approach to introduce both CAR transgenes and CRISPR-Cas9 ribonucleoproteins (RNPs) into primary human T cells using engineered lentiviral particles (84). Additionally, a dual gene therapy strategy has been developed, involving a conditional suicide gene and CCR5 knockout, to overcome limitations associated with CCR5 knockout alone and receptor switching (85).

#### 2.3.1 Cellular and viral targets

The CRISPR/Cas9 strategy targets LTR as one of the most commonly used sites for removing HIV-1 proviral DNA (Figure 3) (86). Moreover, the CRISPR/Cas9 mechanism can also be used to remove regulatory genes of HIV-1, including tat and rev (87). For example, Herskovitz et al. developed a collection of gRNAs targeting the consensus sequence of the HIV-1 viral transcription regulator, tat (88). The integrated HIV-1 provirus is similarly controlled by an internal antisense lncRNA expressed from the nef gene located at the 3′ end of the viral genome, which makes the promoter region in nef a useful target for targeted CRISPR/Cas9 deletion or gene suppression (89). The CRISPR/Cas9 system can inhibit virus replication by targeting host factors and coreceptors, including CCR5 and CXCR4 (Figure 3) (90). In one of these approaches, HSCs are extracted from patients and transfused after CRISPR/Cas9 treatment through deletion in receptor genes. Bogerd et al. used a Cas9-based approach to induce the expression of restriction factors APOBEC3G (A3G) and APOBEC3B (A3B) in human cells (91). The CRISPR knockout screening approach can be used to identify new host genes involved in the virus replication cycle (92).

For the “shock and kill approach,” it was shown that the use of dCas9 aimed at LTR, together with the synergistic activator mediator (SAM) system, allowed to increase the activity of LTR-controlled gene expression. After that, the research team analyzed possible non-targeted effects leading to changes in transcription profiles associated with the use of dCas9-SAM. The authors demonstrated that of the tested genes, only two were significantly activated. Despite the fact that this study was limited by the small size of replication and the fact that further trials in other cell models (and possibly *in vivo*) would be required, this was the first evidence indicating the safety of this strategy for possible future therapeutic applications (Figure 3) (93).

dCas9 fused with the KRAB transcriptional repression domain was adopted to inhibit provirus activation. It was demonstrated that when LRA cells were stimulated, HIV-1 expression decreased by up to 60% compared to the control after delivery of specific gRNAs designed to direct dCas9 to the LTR promoter regions. This effect was associated with the presence of repressive epigenetic modifications, which indicates the possibility of developing a CRISPR system for the “block and block” approach (Figure 3) (94).

#### 2.3.2 Current status

Although off-target effects are detected in all genome editing systems, the high occurrence of unpredictable off-target effects in the CRISPR/Cas9 technology is a serious drawback (95). Other limitations of this strategy include the requirement for a short PAM near the target locus, the complexity of packaging into AAV vectors due to the large size of the most popular, Streptococcus pyogenes, Cas9 (SpCas9), and the fact that CRISPR-induced DSBs often provoke apoptosis, leading to DNA damage and cellular toxicity (96–98). A sterilizing cure approach would require delivery of Cas9 and gRNA to all HIV-1 reservoir cells *in vivo*, but unfortunately, the effectiveness of Cas9 and gRNA delivery appears suboptimal (99). Additionally, immune reactions against the non-human Cas9 protein can complicate this strategy for *in vivo* HIV inactivation (91). Gene therapy and stem cell transplantation are high-cost and need advanced technologies. Nevertheless, recent studies show that the target gene may be gag. Thus, a study by Trisha H. Burda et al. found that SIV-induced rhesus monkeys receiving AAV9 CRISPR-Cas9 and two gRNAs were detected on SIV regions known as EBT-001. It was shown that there are signs of editing of SIV proviral DNA in all major viral reservoirs. EBT-001 was well tolerated in various dosages without obvious toxicity (when taking higher doses, a short-term increase in the level of liver enzymes was observed, which later returned to normal). Overall, this study confirms the potential of AAV9 delivered by CRISPR-Cas9 with dual gRNA, similar to EBT-001, in strategies aimed at eradicating HIV (100).

#### 2.3.3 Supplement on genome editing methods

In recent years, the three main gene editing tools that use nucleases–transcription activator-like effector nucleases (TALENs), zinc finger nucleases (ZFNs), and CRISPR/Cas9–have been widely used in studies on the treatment of HIV/AIDS (83). These methods include inhibiting host factors, weakening transcription and replication of HIV-1, inactivating HIV LTR, suppressing proviral HIV expression, and eliminating latent HIV-1 provirus (4). A previous clinical trial involving 12 HIV-infected patients showed that introducing ZFN-modified autologous CD4 + T cells containing mutated CCR5 is a safe approach to combatting HIV (101). TALENs, in turn, are also site-specific nuclease-based tools used for first-generation genome editing approaches with greater safety than ZFNs (4). According to previous results, CRISPR/Cas9 is a more effective genome editing method for HIV treatment compared to ZFN and TALEN approaches (4, 102). Most CRISPR-based tools have undergone intensive review (103–106). A comparison of different nuclease-based genome engineering platforms has also been conducted (107).

## 3 Antibody based therapies and vaccines

T-cells, including CD4 + and CD8 + cell populations, and B-cell lymphocytes are very important members of the adaptive immune system. During the infectious response, CD8 + T cells are necessary for the direct destruction of infected cells, while CD4 + T cells promote the induction of CD8 + T cells and support the maturation of highly specific antibodies produced from B-lymphocytes (108, 109). B-cells undergo iterative cycles of proliferation, immunoglobulin mutation, and antigen selection for the generation of highly specific antibodies in specialized immune cells in the secondary lymphoid organs (germinal centers or GCs) (70, 108). After an antigen challenge, GCs activate the B-cells by antigen-specific B-cell surface receptors (BCRs) (110). Each GC generally focuses on one specific antigen and can produce a limited amount of antigen-specific B cells (108). This process contrasts with the early B-cell responses to antigens in the extrafollicular spaces that result in short-lived antibody-producing cells (plasmablasts) secreting non-mutated antibodies (70, 111). Eventually, the B-cells depart the GC and might turn out to be either plasmablasts, or memory B-cells (112).

The antibody response to the viral Env, Gag, and Pol proteins, along with detection of p24 protein and viral RNA, can be used for tracking the early stages of HIV-1 progression (113). B-cells respond to HIV-1 infection for the first time within ∼1 week after viral RNA can be detected in the plasma and the immune response is initially observed in the form of virion-antibody immune complexes; subsequently free IgM antibodies to gp41 have been detected (114, 115). Protective neutralizing antibodies (NAbs) develop slowly and do not appear until 8–12 weeks after HIV-1 infection (114, 116). NAbs are produced against viral Env to neutralize the particular viral strain infecting the patient (autologous virus) (117). Only years after HIV-1 infection, could cross-reactive antibodies, able to neutralize heterologous viral isolates, frequently be found (114, 116). HIV-1-specific antibodies can also interact with the Fc gamma receptors (FcγRs) that have the potential to inhibit HIV-1 spread via antibody-dependent cellular cytotoxicity (ADCC), antibody-dependent cellular phagocytosis (ADCP), and antibody-dependent cell-mediated virus inhibition (ADCVI) (116, 118).

In fact, the development of a safe and effective protective vaccine against HIV-1 remains one of the highest priorities for global public health and the best long-term tool for controlling HIV-1 transmission (119). Preclinical and clinical trials have evaluated various approaches to creating HIV-1 vaccines, but the results have generally been unsatisfactory (120). Since vaccines were invented, five basic approaches have been used in the development of viral vaccines; however, the two most effective approaches (attenuated and inactivated organisms) have not been proven optimal for HIV vaccine development (12, 121, 122). Further, HIV vaccine development has shifted direction toward cellular immunity to induce HIV-specific cytotoxic T-lymphocyte (CTL) production. These cells recognize HIV epitopes on the cell surfaces and arrest the proliferation of HIV infection through apoptosis or the secretion of chemokines and cytokines, subsequently interfering with the next rounds of viral replication (12, 70). For instance, one of the approaches to T-cell-based vaccine development involves the induction of non-classical Major histocompatibility complex E (MHC-E) restricted CD8 + T-cell responses by a modified cytomegalovirus (CMV) vector (123, 124). Other approaches to vaccine development have used DNA plasmids and other viruses as vectors to deliver viral genes (lentiviral vectors, integrase-defective lentiviral vectors, recombinant adenovirus type 5 vectors) (12, 125, 126). Although DNA vaccines are safe because DNA plasmids stay episomal and act as expression vectors produced by peptides that can induce cellular immunity, they are not able to induce reliable T-cell levels or antibody responses (12). To boost the immune responses during DNA vaccination, the use of molecular adjuvants is also being explored (127, 128). Some approaches have used mRNA as a vector, also resulting in the induction of polyfunctional antibody responses (70, 127). A vaccine platform of mRNA incorporated into lipid nanoparticles (mRNA-LNPs) has lately been characterized for infectious diseases, notably for SARS-CoV-2 and for HIV (129). The strength of this approach, in addition to the positive aspects of using mRNA (e.g., Env–Gag mRNA designed by Zhang et al. in vaccine development, lies in the induction of both follicular helper cells (Tfh) and B-cells in the GC response (67, 120). Currently, tests are being conducted on various animal models, and the data obtained are hopeful for future use in humans (68, 70). However, it may be years before the most important scientific contribution made by the response to the SARS-CoV-2 epidemic can be applied to HIV vaccine research (130).

**FIGURE 4:**
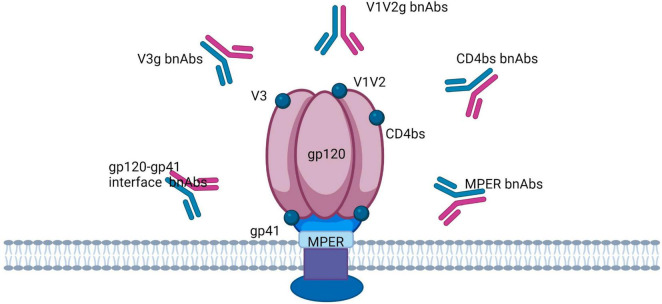
Broadly neutralizing antibodies target the HIV trimer Env in five regions: the CD4 binding site (CD4bs), V1/V2-binding sites, V3-glycan, glycoprotein (gp)41/gp120 interface, and the membrane proximal external region (MPER).

Vaccine development efforts are focused on the induction of neutralizing antibodies (NAbs), in particular broadly neutralizing antibodies (bNAbs) that cover 50–90% of transmitted viruses (131, 132). The serum of a small percentage of individuals (10–30%) living with HIV contains bNAbs, which has provided evidence that a bNAb-inducing vaccine is possible (118, 133). Broad neutralizing antibodies (bnAbs) neutralize multiple HIV-1 strains by targeting conserved epitopes of the virus (Figure 4) (134). The classification of HIV-1 reactive bnAbs includes naturally occurring and engineered antibodies (137). Moreover, there are twenty types of broadly neutralizing antibodies (bnAbs) that have been divided into six categories according to the specific Env residues they interact with. There’s also another class of bnAbs that targets the gp41 membrane proximal external region (MPER) (135, 136). bsAb molecules can also be separated into a class of IgG-like molecules and a class of non-IgG-like molecules (137). Additionally, first-generation antibodies (b12, 2G12, 4E10, and 2F5) and second-generation bnAbs (PG9, PG16, CH01, PGT145, PGT121, PGDM1400, 10-1074, 10E8, VRC01, 3BNC117, and CH103) with improved neutralizing ability and flexibility in technological manipulation have been classified (138, 139).

The first bnAb (b12) was discovered in 1991, and in recent years, there has been an increase in the number (more than 100) of available bnAbs through the introduction of new technologies like Env-specific sorting of individual B-cells, proteomic deconvolution, antibody cloning and high-throughput neutralization assays (118, 138, 140, 141). New technologies enable the construction of antibodies with two, three or four different binding sites on a single molecule (142). Thus, bsAbs are designed either to recognize two different HIV-1 *env* epitopes through the single-stranded variable fragment (scFv) of two independent bNAbs or to interact with cellular receptors with one scFv and one HIV-1 *env* epitope with another scFv. Bispecific (bsAbs) and trispecific bNAbs (tsAbs) represent a promising alternative to bNAb combination therapy because they recognize multiple targets on the viral Env protein (137). Some information about bnAbs is freely accessible on a website known as the Broadly Neutralizing Antibody Electronic Resource (bNAber), which is aimed at motivating researchers to design a reliable HIV vaccine (143). On the other hand, clinical and preclinical studies, for instance, the RV144 vaccine efficacy study, highlighted non-neutralizing antibodies (nNAb) that have no neutralizing activity *in vitro*, but may play an important role in protecting against viral infection *in vivo* (123, 124). Several human monoclonal antibodies with broadly neutralizing activities have been observed (F105, b12, 2F5, and 4E10) that are specific to the CD4^+^ T-cell binding site on gp120 or gp41 and demonstrate antiviral protection against different HIV clades (A, B, C, and D) *in vitro* or in experiments with neonatal macaques (144, 145). BG505 SOSIP is a well-studied, almost native recombinant HIV envelope trimer (Env) that holds promise as part of a successive anti-HIV immunogenic scheme for the induction of bnAbs (123, 146).

### 3.1 Viral and cellular targets

Effective HIV-1 vaccines depend upon T-cell-mediated immunity (CMI) focused on rather conserved viral proteins, which include Gag and Pol and/or non-neutralizing antibodies targeting the virus envelope, to prevent cell-to-cell transmission of the virus (147, 148). HIV-1 virions contain about 10–14 trimeric envelope glycoproteins (Env) on its surface, which mediate the penetration of the virus into host cells (149). Every HIV envelope spike protein (Env) comprises three external gp120 subunits that are non-covalently bound to three gp41 subunits attached to the membrane (Figure 4) (128). The Env glycoprotein is strongly glycosylated, and a dense shell of host-derived N-glycans protects the epitopes of the viral protein from antibody interactions (150). The new antibody group has been helpful in identifying a highly structured epitope present only on the trimeric envelope and including the conserved V2 and V3 regions that are symbolically represented in Figure 4 (116). Xu and group designed highly potent trispecific antibodies by combining the specificity of PGDM1400, VRC01, and 10E8v4 to interact with the membrane-proximal external region (MPER)-, CD4-, and V1/V2-binding sites (Figure 4) (151). bnAbs are thought to break through the glycan protection of the HIV trimer *env* in five regions, each of which is probably involved in the *env* function: the CD4 binding site (CD4bs), the variable loop 2 (V2)-apex, the V3-glycan, the glycoprotein (gp)41/gp120 interface, and the membrane proximal external region (MPER) (Figure 4) (152–154). It is believed that the apical region V2 is participating in maintenance of the metastability of the spike Env protein (155). The V3 glycan site is formed partially by the co-receptor site CCR5 and partially by the surrounding masking glycans (156). The interface region for glycoproteins gp120 and gp41 includes the fusion peptide (FP) and the cleavage site of gp160 into gp120 and gp41 (152, 157). In turn, MPER is part of the fusion machinery (152, 158).

### 3.2 Restrictions

Firstly, it is improbably that the HIV vaccine will be sufficient to stop a persistent HIV-1 infection (12). Secondly, HIV infection gradually disrupts the body’s immune response, which is necessary for the effectiveness of the vaccine (159). The ever-changing antigenic variations of HIV represent the third major problem for vaccine development (139). For, instance, based on whole-genome sequences, HIV is classified into three major groups: major (M), outlier (O), and non-M/non-O (N), and their prevalence varies according to geographical regions (12). Nine of the HIV subtypes belonging to group M form HIV subtypes or “clades” (A–D, F–H, J–K), which differ by ∼25–35% in their *env* sequences and ∼15% in their *gag* sequences (12, 123, 160). Moreover, the evolution of HIV-1 in infected individuals begins shortly after infection (123). New approaches to creating multivariate vaccines will probably be needed. The development of antibody production techniques is also strongly linked to the cost (138, 161). Other key difficulties are the delivery and stability of antibodies for HIV therapies.

## 4 Cell therapies

T-lymphocytes in the immune system can identify invading pathogens when they’re exposed to the pathogen’s specific immunogenic component, known as an antigen (162, 163). T-cell activation during infection happens through the T-cell receptor (TCR), which includes the CD3 receptor and either the CD4 or CD8 co-receptors. The CD4 receptor is utilized by helper T-cells to activate their ability to release cytokines, while cytotoxic T-cells use the CD8 receptor to enhance their capacity to kill cells (164). Once activated, T-cells start to multiply quickly, but after the infection is resolved, the population of activated T-cells, which was formed by clonal expansion, reduces and ultimately undergoes apoptosis (165). After this phase, a small group of memory cells remains. These cells can recognize the same antigen, quickly expand clonally, and differentiate to trigger a powerful and specific adaptive immune response (143, 151, 166). During ripening in the thymus, T cells that recognize their own peptides are destroyed to prevent autoimmune reactions (167). Additionally, the cytotoxic T-lymphocyte (CTL) response is a crucial aspect of host immunity against HIV infection (168, 169). It is generally believed that in elite controllers (a rare set of individuals who can control HIV replication for extended periods without anti-HIV treatment), the control of the virus is mostly mediated by the CD8 + T-cell response (170–172). CTLs also have the ability to direct the lysis of infected cells via major histocompatibility complex class I (MHC-I) molecules. However, HIV can potentially decrease the surface expression of MHC-I on infected cells to avoid this immune response (173, 174).

**FIGURE 5:**
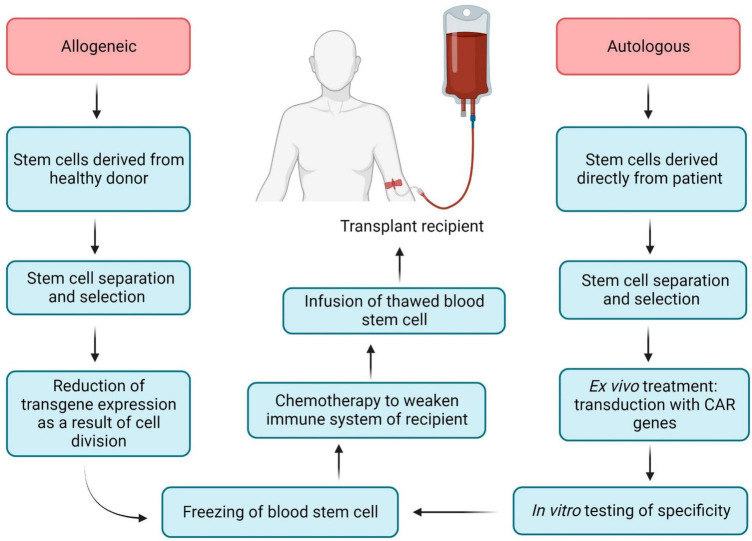
Allogeneic and autologous blood stem cell transplantation.

Chimeric antigen receptor (CAR) is an engineered TCR known to bind with a specific antigen, and after introducing CARs into T-lymphocytes, CAR T-cells can be obtained. The CAR transgene is a synthetic chimeric receptor comprising an scFv of the antibody and T-cell signaling domain(s), whereas another approach includes using a TCR transgene derived from a native TCR that has been partially modified to increase its affinity for the target antigen (Figure 5) (175). For most CAR T-cell therapies, cells are extracted from the peripheral blood mononuclear cells (PBMCs) of the patients by apheresis or leukapheresis (164). This sample represents a diversity of immune cells, including B-cells, macrophages, monocytes, natural killer (NK) cells, and T-cells. The first stage of preparation requires the identification of a subset of T-cells from the PBMCs, and this can be achieved by magnetic bead selection or selective expansion (176, 177). Anti-CD3 or a combination of anti-CD4 and anti-CD8 antibodies can be used for T-cell selection (178). Activated T-cells are then grown in culture to achieve the required number of cells and transduced with the CAR cassette (179). Gene delivery can be accomplished by stable integration of the cassette into the genomic DNA of the host T-cell assisted by viral vectors (e.g., lentiviral vectors) and by non-viral transfer or non-integrating transient delivery (180). Afterward, cell material is collected and cryopreserved at -120°C, and then the sample is returned to the clinic for intravenous infusion into the patient (Figure 5) (164). As an example, CD8 + T cells are harvested from HIV patients and transduced with CAR genes; after testing specificity and efficacy against HIV *in vitro*, functional HIV-specific CAR T cells are re-injected into patients to destroy HIV-infected cells (17).

Hematopoietic stem cells (HSCs) perform two important functions: their ability to self-renew and the ability to differentiate into various hematopoietic lines, including lines that have the ability to kill HIV-infected cells (T-cells and NK-cells) (181). Hematopoietic stem cell transplantation (HSCT) has become a promising candidate for achieving a functional cure for HIV, mainly due to the clinical experience of the Berlin patient and the London patient (182–184). Both patients received HSPCs from donors with a naturally occurring 32 base pair deletion in the CCR5 gene corresponding to the second extracellular loop of the receptor (CCR5Δ32), resulting in a non-functional gene product that is not expressed on the cell surface due to frameshift and early termination (185, 186). The stable viral remission in these patients after cART interruption is presumably explained by a combination of a conditioning regimen that provided donor chimerism, destruction of host latent reservoirs during donor cell engraftment and almost full substitution of the host immune system by homozygous CCR5Δ32 donor cells (187–189). While the positive outcomes of these two patients’ therapies constitute a significant landmark in the effort to cure HIV, this method includes several limitations (risk of morbidity and mortality, limited prevalence of CCR5Δ32 donors, HIV mutation via a CCR5- to CXCR4 tropism shift) (16, 190). These limitations make allogeneic HSCT unfeasible for the vast majority of people living with HIV (187). HIV-specific CAR expression from genetically modified autologous HSPCs has the potential to bypass the limitations of the allogeneic HSCTs. Previously CD4ζ based CAR-modified HSPCs were differentiated into functional T-cells as well as NK cells *in vivo* in humanized mice, moreover, these cells are HIV-resistant (191). In addition, NK-cells can identify and eliminate HIV-infected signals using the mechanism of antibody-dependent cell-mediated cytotoxicity (ADCC) (191, 192). Novel genetically modifying HSPCs to express CD4 CAR are long-lived and proliferate in multiple tissues relevant to HIV infection and cancer (lymphoid germinal centers, brain, and gastrointestinal tract) for almost 2 years and have demonstrated multiphasic engraftment in macaques (187). All commercially accessible adoptive T-cell therapies have been autologous (cells derived directly from the patient), as the allogeneic approach has been shown to be complex to design through early HLA typing and stem cell technology (193).

**FIGURE 6:**
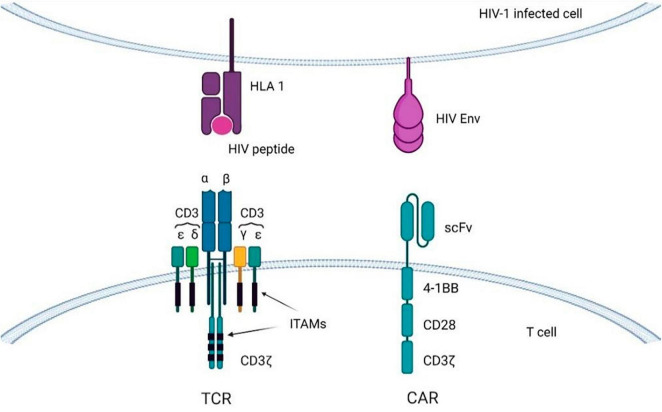
T-cells with CARs or transgenic TCRs targeting viral antigens displayed on HIV-infected cells. Immunoreceptor tyrosine-based activation motifs (ITAMs) are signaling motifs for adaptive immune responses regulation. CAR, chimeric antigen receptor; TCR, T-cell receptor; HLA, human leukocyte antigen.

In early clinical trials the first generation of CAR constructs had a single intracellular signaling domain from the CD3ζ of the TCR, fused to either the extracellular CD4 region (CD4ζ-CAR), or to the scFv of isolated monoclonal antibodies (scFv-CAR) (6, 181, 194). The second generation also has a CD28 signal, which promotes cell proliferation and cytokine secretion (195). The third generation of CARs supplemented the 4-1BB and OX40 regions to favor cell survival and to extend the persistence of CAR T-cells *in vivo* (Figure 6) (196). The latest generation, recognized as T-cells redirected for Universal cytokine-mediated destruction (TRUCKs), has been developed lately and carries specific cytokine signals that make CAR T cells resistant to the immunosuppressive effects of the tumor microenvironment (TME) (196, 197). However, when employing a panel of HIV-specific (CD4-based) CARs expressing distinct intracellular domains (ICDs) it was shown that only HIV-resistant, 4-1BB-stimulated CAR4 T-cells restrict HIV infection *in vivo* (198).

The 2016 study included 28,992 patients. The average number of CD4-T cells at the beginning of treatment was 249 cells/ml. The average observed CD4 count after 6, 9, and 12 months was 382, 402, and 420 cells/μl. The two main factors explaining the change in CD4 count after 6 months were the stage of AIDS and the CD4 count at the beginning of cART. The median observed CD4 count at 6, 9, and 12 months was 382, 402, and 420 cells/μl. The two main factors explaining the change in the number of CD4 after 6 months were the stage of AIDS and the number of CD4 at the beginning of cART (199).

Additionally, with the discovery in the last few years of many potent bNAbs against HIV, CAR T therapy relying on bNAbs is thought to be a promising strategy for the treatment of HIV infection (200). Direct comparisons between bNAb- and CD4ζ-based CARs, or between bNAbs, have illustrated certain distinctions in breadth and potency and indicate that at least some antibody-derived scFVs may be more suitable than others for CAR T-cell administration (194).

### 4.1 Viral and cellular targets

Compared to CAR-T cells targeting tumor antigens such as CD19 and CD20, both of those are similarly expressed in healthy tissues, HIV-1-specific CAR-T cells target HIV-1 *Env*, that is expressed only on the surface of virus-producing cells (200). There are also two shared strategies for targeting HIV, one of which involves CD4 to target cells expressing HIV *Env*, while the second strategy involves aspects to prevent HIV infection of CD4-based CAR-T cells (6). Scholler et al. stated that, in general, CD4 receptor-based CAR T-cells exhibit a half-life of longer than 16 years with a stable engraftment level and safety (201). A recent study has demonstrated that primary T-cells transduced with a multispecific CAR (targeting the CD4-binding site gp120 and the co-receptor gp120 binding site) can potently inhibit cellular HIV infection by up to 99% *in vitro* and >97% *in vivo* (17, 202). To avoid identification of CD4-CARs HIV-1 as target cells, CD4-CARs are engineered to co-express HIV-1 fusion inhibitors, such as membrane-bound peptide C or peptides from the heptad repeat-2 domain of gp41 (203, 204). Alternative constructs have been developed for co-expression of shRNA targeting CCR5 and the LTR sequence to inhibit HIV-1 entry and to facilitate viral RNA degradation (205). The other strategy for avoiding infection of CD4ζ CAR T-cells is to modify the antigen-recognition part of the CAR molecule (181). Also, *in vitro* data showed that V1/V2-specific cars outperform CAR targeting CD4bs glycan and V3 on Env SIV. The transfer of anti-SIV CAR T cells to SIV-infected animals gave neither protection nor viral control. Unlike CAR-T-cell cancer therapy, CAR therapy against HIV requires a greater improvement in viral control.

Despite the recent FDA (Food and Drug Administration) approval of CAR-T cell therapy for B-cell leukemia and extensive research efforts in the field of cancer immunotherapy, about seven clinical trials of CAR-T cells against HIV have been initiated, five of which are currently in active status (NCT03240328, NCT04648046, NCT03617198, NCT01013415, NCT05077527), one completed (NCT03980691) and one in status unknown (NCT04863066) while clinical trials have previously confirmed the safety and efficacy of CAR-T cells against HIV validity of carcinotherapy.^1^

### 4.2 Restrictions

T-cells with CARs against the HIV envelope can increase the adaptive immune response, but are ineffective in controlling viremia (194). Another possible obstacle to CAR-therapy of HIV infection is an extremely low level of expression of the viral envelope on the surface of HIV-infected cells, moreover, the development of CAR-T-cell therapy is associated with a number of problems, including weak cell expansion, short vitality *in vivo* and significant side effects in patients (cytokine storms and neurological toxicity) (206). Strategies for improving the function and persistence of CART cells have been thoroughly researched for CARS based on CD19 and other tumor-specific antigens. In addition, research supporting the concept has shown the HSPCs are capable of lifelong engraftment and ensure the proper development of CAR-T cells *in vivo* (191). Although stem cell-based CAR-T-cell therapy against HIV has proven to be possible and effective in a humanized mouse model, there are limitations in the use of this model, which include a deficiency of the lymphoid structure and a graft vs. host reaction (207). Difficulties with the application of the CAR antibody are related to possible immunogenicity and the design of anti-idiotypic antibodies that can inhibit their activity (208). There is a risk that CAR may likewise attack healthy cells expressing the same or identical target antigen, that is recognized as an “off-target” effect (17).

## 5 Eradication of latent reservoirs

### 5.1 Establishment of HIV-1 reservoirs

Latent HIV reservoirs are the central barrier preventing a HIV-1 cure. A large number of cell types, including CD4 + T-cells, macrophages, and dendritic cells, are susceptible to HIV-1 infection, but CD4 + memory T-cells (primarily memory T-cells or TCMs and transient memory T-cells or TTMs) are widely considered as typical latent reservoirs for HIV-1, because of their active and resting physiological state, and the dynamic process of transforming effector cells into memory cells (209–211). HIV-1 reservoirs share a broad spectrum of anatomical localizations, including the lymph nodes, gut-associated lymphoid tissue (GALT), liver, genital tract, and brain (212). For instance, astrocytes and microglia are considered to be macrophage-like cells in the central nervous system (CNS) that are possible reservoirs of viruses in the brain (192). Viral DNA has also been found in HSCs from patients undergoing cART, which may indicate that these cells are involved in the persistence of HIV-1 (213). Some latent regions are shielded from cART penetration (the brain, testicles, and lymph node B-cell germinal centers) and present additional challenges to HIV treatment (214, 215). There is a lot of data from *in vitro*, *in vivo*, and animal models, but there is still no gold standard for defining the size of the latent reservoir (216). However, along with other techniques, digital droplet PCR assays can now detect intact, cell-associated, full-length genomic HIV DNA with increased sensitivity (217–219).

Human immunodeficiency virus type 1 reservoirs are a highly heterogeneous pool of infected cells that can be in different states of viral activation: (a) deep latency, in a state where no viral RNAs are expressed, (b) low transcriptional activation, when small amounts of viral RNAs are produced but not translated, or (c) dynamic viral activation, in this case there is a high level of expression of HIV-1 RNAs and a proportion of these RNAs are later translated into protein (193). The maintenance of the latent reservoir exists via clonal expansion of HIV-infected cells or through infection of long-term reservoir cells with both intact and defective proviruses (212, 220). It is suggested that the expansion of latently infected cells could be propelled by survival advantage and homeostatic cytokines such as IL-7. Moreover, latently infected CD4 + T-cells with antigen-specific TCR can divide in response to recurring exposure to antigens (220). Two primary models are proposed to clarify the latent infection of memory T-cells: the pre-activation and post-activation latency models (216). The pre-activation latency model posits that resting CD4 memory T-cells are infected with HIV-1 before being reactivated by environmental stimuli. However, this model might be inefficient due to the instability of the pre-integration complex, characterized by non-integrated linear and cytoplasmic forms of the viral genome with a half-life of about 1–6 days (221–223). The post-activation model suggests that activated CD4 T-cells are infected by HIV-1 while returning to a resting state, which results in the integration of the proviral genome into the host cell. This avoids creating favorable conditions for optimal viral gene expression, preventing the quick eradication of the infected T-cell (223, 224). Nevertheless, recent research implies that the relationship between T-cell activation and HIV-1 latency might not be as strongly correlated as previously assumed (216). This perspective proposes an additional model, suggesting that HIV-1 latency established in activated CD4 T-cells shortly after infection ensures greater survival capacity and possibly a return to a resting memory state. This process may facilitate the creation of long-lived latent reservoirs (224).

The selection of the HIV-1 integration site may contribute to the establishment of HIV-1 latency mechanisms in activated CD4 T-cells. HIV enters the host and replicates locally at the site of entry, then HIV rapidly circulates to the lymph nodes (within a few days) and further (within a few weeks) into the bloodstream (225). HIV-1 integration appears to be random, but prefers the introns of transcriptionally active genes found in gene-dense regions of the nuclear outer envelope near the nuclear pores (213). For example, pyrosequencing was used to identify 40,569 integration sites in Jurkat cells and another study revealed 6,719 integration sites in CD4 T-cells in a research study involving 13 individuals (226–228). The latency of HIV-1 is controlled by various related mechanisms acting at the transcriptional and post-transcriptional levels, and depends on the transcription program of the host cell (229, 230). In the latent period, the HIV-1 promoter is largely controlled by epigenetic mechanisms, including DNA methylation and post-translational histone modifications such as acetylation, methylation and crotonylation (213). Additionally, there are cellular cofactors of HIV-1 integration such as lens epithelium-derived growth factor (LEDGF/p75) (231).

The other possible interpretation of latency can be derived from the theory of stochastic gene expression. According to this theory, random mutations in a critically important HIV-1 Tat region can inhibit active HIV-1 transcription independently of the target cell activation (232, 233). The majority of investigations on this subject have been performed *in vitro*, which leaves a lot of questions about the level to which the different triggers contribute to the establishment of latency *in vivo* (216).

Strategies for the functional treatment of HIV, based on the “block and lock” approach, aim to induce transcriptional gene silencing (TGS) using latency-promoting agents (LPAs). This approach intends to block viral replication and lock the viral promoter into a dormant state through repressive epigenetic modifications (212). Epigenetic silencing can be initiated through various RNAs molecules (such as siRNAs, shRNAs, and lncRNAs) and small molecule inhibitors (including LEDGIN, epigenetic reader bromodomain and the extraterminal (BET) family proteins BRD4, Torin1, and pp242) (234–237). RNA-directed TGS results in the attraction of additional proteins in the nucleus, forming the RNA-induced transcriptional silencing (RITS) complex. This complex leads to the increase of repressive epigenetic markers such as histone and CpG methylation and the reduction of histone acetylation at the promoter (212).

The other strategy is the “shock and kill” approach when the latent HIV is reactivated by latency reversal agents (LRAs) followed by eradication of the cells with reactivated virus, achieved by enhancing the cytotoxic effect, immune clearance and additional procedures (210, 242). In these studies, the first generation of LRAs successfully induced viral RNA production, but only certain agents were able to cause the protein and viral particle generation (220). The next generations of LRAs in preclinical data demonstrate that small molecule antagonists of apoptosis (second mitochondria-derived activator of caspase or SMAC mimetic compounds) trigger the reversal of the latency (243). A number of LRAs have been developed based on *in vitro* and *ex vivo* systems, including HDAC inhibitors (HDACis), histone methyltransferases inhibitors (HMTis), and DNA methyltransferase inhibitors (DNMTis) (210). Immunostimulatory approaches based on Toll-like receptor (TLR)-7 agonists have displayed direct latency reversal activity in non-human primates, but their efficacy has not been confirmed in subsequent studies (244, 245). A further class of LRAs includes compounds that modulate protein kinases in signaling pathways upstream of the transcription factors that bind the LTR (246). Cytokines and LRA compounds can also be used in combination with recombinant macromolecules (CRISPR/nuclease deficient Cas9 (dCas9) and zinc finger proteins) to reverse HIV latency (247). The main LPAs and LRAs are summarized in Table 1.

**TABLE 1:** The main latency reversing agents (LRAs) able to eradicate the latent HIV-1 reservoirs and latency promoting agents (LPAs) to make the proviral state of HIV-1 deeper.

Latency-promoting agents (LPAs)	
siPromA	(218–220)
ASP RNA	(221)
lncRNA NRON	(222)
Small molecule inhibitors (LEDGIN, BET family proteins BRD4, Torin1, and pp242)	(223–225, 230)
dCA	(238)
**Latency reversing agents (LRAs)**	
Histone deacetylase inhibitors (HDACis), Histone methyltransferases inhibitors (HMTis), DNA methyltransferases inhibitors (DNMTis)	(189)
Immune checkpoint inhibitors	(226, 235–237)
lncRNAs HEAL and MALAT1	(41, 74, 239)
SMAC mimetic compounds	(232)
Toll-like receptor (TLR)-7 agonists	(240, 241)
Protein kinase stimulators, Cytokines, CRISPR/Cas9, ZFN	(233)
TLR agonists	(241)

In persistent viral infections, the large volume of antigens continually stimulates T-cells, leading to a gradual loss of functionality known as T-cell exhaustion (248). During this phase, there is an increased expression of immune checkpoint molecules (ICs) on T-cells. These molecules include programmed cell death protein 1 (PD-1), cytotoxic T-lymphocyte-associated protein 4 (CTLA-4), lymphocyte activation gene 3 (LAG-3), T-cell immunoglobulin and immunoreceptor tyrosine-based inhibitory motif domain (TIGIT), T-cell immunoglobulin and mucin domain 3 (TIM-3), CD160, and 2B4 (CD244) (19). IC expression leads to suppression of the immune response and serves as a marker for HIV latently infected cells with a higher tendency to viral transcription (249). Immune checkpoint blockade in HIV has been intensively studied (250, 251). For example, PD-1 or Interleukin-10 (IL-10) blockade have been shown to reactivate CD4 + T-cell function *in vitro* and to restore NK-cell support (252).

### 5.2 Viral and cellular targets

Latency-promoting agents target the NF-κB sites, the interaction complex of LEDGF/p75 and HIV integrase, Tat, and the mammalian target of rapamycin (mTOR) signaling pathway as an important modulator of HIV-1 latency, triggering recruitment of chromatin-remodeling complexes, including DNA methyltransferase 3 alpha (DNMT3a), histone-lysine *N*-methyltransferase enzyme (EZH2) and histone deacetylase 1 (HDAC-1) (212). For instance, SiPromA was identified in 2005 as the first anti-HIV agent to induce TGS, because it targeted NF-κB sites in the HIV-1 promoter (253–255). Further, HIV-1 encoding antisense protein (ASP) was identified. ASP RNA recruits the repressive Polycomb group 2 complex (PRC2) to the 5′ LTR HIV-1 promoter, resulting in repressive epigenetic modifications (an increase in H3K27me3 marks and a decrease in RNA polymerase II occupancy) (212, 256). Moreover, an lncRNA named NRON was found in resting CD4 + T-cells suppressing viral transcription by causing degradation of Tat (248). However, the virus promoter can be activated by lncRNA HEAL and lncRNA MALAT1 (257–259). McBrien et al. used a provocative method to activate HIV-1 provirus. They used the drug N-803 to induce IL-15, a protein that promotes viral transcription, and an antibody to deplete the CD8 + T-cells that appear to play a role in stabilizing viral latency (260).

### 5.3 Restrictions

A major barrier to HIV-1 eradication is the multiplex mechanism of establishing HIV latency, and the way the latent reservoir recovers and produces infectious HIV virions when ART is terminated (228). Targeting and reactivating latent cells is problematic because of the highly heterogeneous nature of the viral reservoirs. In addition, some studies indicate controversial effects of LRA on NK cell function and on cytotoxic T-cell lymphocytes (CTLs) (213). Escape mutations in dominant CTL epitopes prevent the targeting of induced cells and certain LRAs suppress CTL function (261). The eradication strategy requires better LRA penetration into the tissues by improving the drug delivery system and, most importantly, enhancing the killing of LRA-activated cells by stimulating the CD8 + T response (194). In addition to low efficacy in the clinic, other disadvantages of many LRAs are their adverse effects and toxicity (237).

## 6 Conclusion

According to the latest update (March 2022) from the U.S. National Institutes of Health (see text footnote 1), 630 clinical trials associated with HIV-1 therapy have been initiated. Although some clinical trials have shown that gene-based therapeutic approaches in combination with conventional therapies can eliminate the HIV-1 virus, most gene-based clinical tests are still in the early stages (4). All mentioned advantages and limitations of HIV-1 cure strategies have been included in Table 2.

**TABLE 2:** The main advantages and limitations of HIV-1 cure strategies.

HIV-1 cure strategies	Advantages	Limitations	References
RNA interference	Small size and low capacity to trigger adaptive immune responses	RNAi side effects; off-target events; poor stability; insufficient delivery	(22, 27, 46, 51)
Antibodies	Reduced toxicity; Potency for the activation of a broad immune response against HIV-1	Delivery and stability; low-efficiency; high cost	(69, 80, 127, 238)
Cell therapy	Capability of destroying latently infected cells	Poor cell expansion; short *in vivo* viability; side effects	(148, 158, 163)
Latency reversing agents and latency promoting agents	Targeting and reactivation of latent HIV-1 reservoirs	Delivery system; Low efficacy in the clinic; adverse effects and toxicity	(194, 230)
Genome editing	Reduction of proviral sequences	Off-target effects; large size and PAM requirement for Cas9; non-optimal delivery; adverse immune reactions; expensive technologies	(249–252, 258, 259)

As with cART, strategies combining multiple antiviral approaches should be considered to avoid the escape of HIV-1 mutants (Table 3). Although, there is evidence that viruses have developed mechanisms to escape the RNAi defensive mechanism, RNAi-based therapeutics can be enhanced by using a combination of different siRNAs or by coupling the siRNAs with ribozymes, aptamers and antiviral proteins (such as RevM10) (28). Strand selection can be biased by constructing asymmetric siRNAs or by chemical modifications at one or both ends of the siRNA (4). Dicer-independent Ago/shRNAs have the potential to demonstrate an improved safety profile and to reduce off-target effects compared to conventional shRNAs, but identifying their overall benefit requires additional laboratory testing (60, 262, 263).

**TABLE 3:** Combinatorial strategies for functional treatment of HIV-1.

Combination of different siRNAs or siRNAs with ribozymes, aptamers and antiviral proteins (such as RevM10)	(24)
Latency re-activators in CAR T-cell therapy	(183)
Combined or bispecific CARs	(164, 165)
Combination of CCR5 gene editing, bNAbs and CAR T-cells	(149, 180)
The combined use of LRAs	(63, 264)
Combination of LRAs	(265, 266)
LRA combinations with vaccines	(267, 268)
CRISPR-Cas9 with antiviral drugs or RNAi molecules	(258)

To date, the accumulated data indicate that no single factor will define the ultimate achievement of a bNAb-inducing HIV-1 vaccine, that probably requires a combination of effective priming of B-cell precursors, optimization of *Env* design and presentation, as well as sustained enhancement of heterologous *Env* (120). To resolve the problems of antibody delivery and stability, antibodies may be conjugated with cell-penetrating peptides (CPPs) derived from various sources to penetrate the cell cytoplasm (269). Smaller antibodies such as Fabs, scFvs and single-domain antibodies or sdAbs (with Fc removed) have also been created for this goal (138, 270, 271). Modifications that increase the half-life, potency, Fc-receptor (FcR) binding, and polyfunctionality are thought to bypass the several disadvantages of bnAbs (272).

Combined or bispecific CARs may be necessary to overcome the well-documented capacity of HIV-1 to mutate and escape treatment or host immune responses, for example, bispecific CARs have displayed improved efficacy against several primary HIV-1 isolates compared to single CD4ζ CARs and this approach deserves additional *in vivo* studies (273, 274). CAR approaches for people with HIV may be improved with a combination of therapies, such as CCR5 gene editing, the use of individual bNAbs targeting various regions of the viral envelope and the development of next-generation CAR T-cells capable of acting on multiple antigens (181, 191). The incorporation of co-stimulatory domains, including CD28, 4-1BB, CD28 + 4-1BB, OX40, ICOS, and CD27, or the engineering of CD4-ζ CARs in second- and third-generations could increase the proliferation and killing efficiency of these cells (181, 275, 276). In addition, the use of latent re-activators in CAR-T cell therapy could potentially allow CAR-T cells to act on latent reservoirs, since these CAR-T cells are able to move into various types of tissue reservoirs, including the central nervous system, which is a potentially significant refuge for latent HIV (276).

The combined use of LRAs with synergistic effects is currently an actively studied area of research (9, 172). Combined approaches, which include LRAs with several different types of mechanisms, are being studied to obtain more effective shocks (277, 278). HIV reservoirs are often hidden in sites, such as lymphatic, gut or brain tissues, but the development of nanoparticle-packed cART drugs or CRISPR-Cas9 system have the ability to directly target the provirus and to destroy the HIV reservoirs (265). LRA combinations with vaccines targeting conservative HIV-1 epitopes have also generated interesting results (266, 279).

CRISPR-Cas9 can be combined with other anti-HIV therapies (antiviral drugs or RNAi molecules) to reduce viral replication, but these combinations also increase the genetic threshold at which viral escape can occur (99). Current efforts are focusing on reducing the number of CRISPR off-targets by creating alternative Cas9 variants (SaCas9, Cas12a, Cas13a, Cas13d, base editors and prime editing systems) and improving the architecture of gRNAs (239, 280, 281). Moreover, non-viral delivery systems also have been extensively investigated and reviewed (63, 240, 264, 267, 268).

Eventually, the high initial cost of cell and gene therapies will become more cost-effective than conventional cART, if a single treatment can be sufficient (31). Combined cell and gene therapies have come a long way, and their great potential will open up new opportunities for the development of HIV cures.

## Author contributions

AS: Conceptualization, Data curation, Formal analysis, Investigation, Methodology, Validation, Visualization, Writing – original draft, Writing – review and editing. EA: Conceptualization, Data curation, Formal analysis, Investigation, Methodology, Validation, Visualization, Writing – original draft, Writing – review and editing. ED: Conceptualization, Funding acquisition, Project administration, Validation, Writing – review and editing.

## Footnotes

1
https://www.clinicaltrials.gov
